# Gold cauldrons as efficient candidates for plasmonic tweezers

**DOI:** 10.1038/s41598-020-76409-3

**Published:** 2020-11-09

**Authors:** Mohammad Ali Khosravi, Abolfazl Aqhili, Shoaib Vasini, Mohammad Hossein Khosravi, Sara Darbari, Faegheh Hajizadeh

**Affiliations:** 1 Nano Plasmo-Photonic Research Group, Faculty of ECE, Tarbiat Modares University, Tehran, 14115-111 Iran; 2 Nano Plasmo-Photonic Research Group, Faculty of Engineering, Tarbiat Modares University, Tehran, 14115-111 Iran; 3 Department of Physics, Institute for Advanced Studies in Basic Sciences (IASBS), Zanjan, 45137-66731 Iran; 4 Optics Research Center, Institute for Advanced Studies in Basic Sciences (IASBS), Zanjan, 45137-66731 Iran

**Keywords:** Optics and photonics, Optical techniques, Optical manipulation and tweezers

## Abstract

In this report, gold cauldrons are proposed and proved as efficient candidates for plasmonic tweezers. Gold cauldrons benefit from high field localization in the vicinity of their apertures, leading to particle trapping by a reasonably low power source. The plasmonic trapping capability of a single gold cauldron and a cauldrons cluster are studied by investigating the plasmon-induced variations of the optical trap stiffness in a conventional optical tweezers configuration. This study shows that the localized plasmonic fields and the consequent plasmonic forces lead to enhanced trap stiffness in the vicinity of the cauldrons. This observation is pronounced for the cauldrons cluster, due to the additive plasmonic fields of the neighboring cauldrons. Strong direct plasmonic tweezing by the gold cauldrons cluster is also investigated and confirmed by our simulations and experimental results. In addition to the presented plasmonic trapping behavior, gold cauldrons benefit from a low cost and simple fabrication process with acceptable controllability over the structural average dimensions and plasmonic behavior, making them attractive for emerging lab-on-a-chip optophoresis applications.

## Introduction

Ashkin is well-known as the pioneer of using light for optical manipulation for the first time^1,2^. The concept of optical manipulation is based on momentum transferring from an electromagnetic field to an object and is known as optical tweezers (OT), wherein a Gaussian light beam is highly focused to the scale of the target object^3^. Although there are numerous reports that have demonstrated successful manipulation of micro-particles^4,5^, optical manipulation of nano-particles is still challenging because of the diffraction limit in OT. To overcome this challenge, incident laser power can be raised to enhance the exerted optical forces on the nano-particles^5^, which may lead to damage the sensitive target particles, such as biological samples^6^. The other drawback of OT is the bulky external optical equipment of light focusing, which hinders the size reduction of the related optophoresis systems and the ability to realize lab-on-a-chip (LOC) devices. On the other hand, plasmonic tweezers, benefiting from inherent highly confined and strongly enhanced local electromagnetic field, has been attracted numerous researchers for trapping, sorting, separation and sensing of particles^7,8^. Plasmonic tweezers take advantage of the strong plasmonic field gradient at the vicinity of the metal/dielectric interface, leading to a strong gradient force at a lower laser power and without the need for light focusing equipment. In this line of research, there are different plasmonic tweezers reports, using different metallic structures, such as nanodiscs^9,10^, nano-holes^11–14^, nano-pillars^15^, nano-pyramids^16^, nano-triangles^17–19^, micro/nano-stripes^20^, micro/nano-rings^21^, as well as graphene-based structures^22–24^. Among these structures, nano-holes^25,26^ and nano-discs have attracted great interest, in many plasmonic applications, including plasmonic tweezers. However, it has been proved that plasmon-induced heat generation at the hot spots of theses metallic structures have negative effect on the stable trapping of the sensitive target particles, the drawback which is more pronounced in nano-disc arrays in comparison with nano-hole arrays, due to their lower heat transfer from the hot spots^27^.

Despite of numerous reports on one and two dimensional plasmonic structures, 3D plasmonic structures^28,29^ have been rarely reported, especially for plasmonic tweezers applications. Herein, we have made gold cauldrons, as a 3D plasmonic structure, by microsphere lithography method, which is a simple, low cost, and large area fabrication process with acceptable controllability^30^. Also, we show that these 3D cavity-like plasmonic structures benefit from large evanescent fields, suitable for particle trapping. On the other hand, Sainidou et al. have previously reported that 3D cavity-like plasmonic structures have the potential of optically tunable trapping of metallic targets^31^, the attractive aspect that can be pursued for the presented gold cauldrons, and can lead to emergence of optically tunable plasmonic tweezers. Moreover, similar to metallic nano-holes, the proposed gold cauldrons can benefit from acceptable heat transfer from the plasmonic hot spots. We believe that the other potential advantage of the proposed gold cauldrons over the previously reported plasmonic structures is their ability for mechanical trapping of target particles because of their cavity-like structures. This feature can be beneficial for confining the Brownian motion and better analysis of the nanoscale target particles, such as proteins, quantum dots, or viruses, based on their acoustic vibrations^32^.

In this report, we have investigated the plasmonic characteristics of the achieved gold cauldrons, and their plasmonic trapping behavior. For this purpose, we utilized the gold cauldrons in a conventional optical tweezers configuration and studied the modulation of the optical tweezers stiffness as a result of the plasmonic forces. Finally, we prove successful plasmonic trapping of polystyrene (PS) particles with radius of 500 nm, utilizing the presented gold cauldrons as the plasmonic structures.

## Fabrication process and results

**Figure 1:**
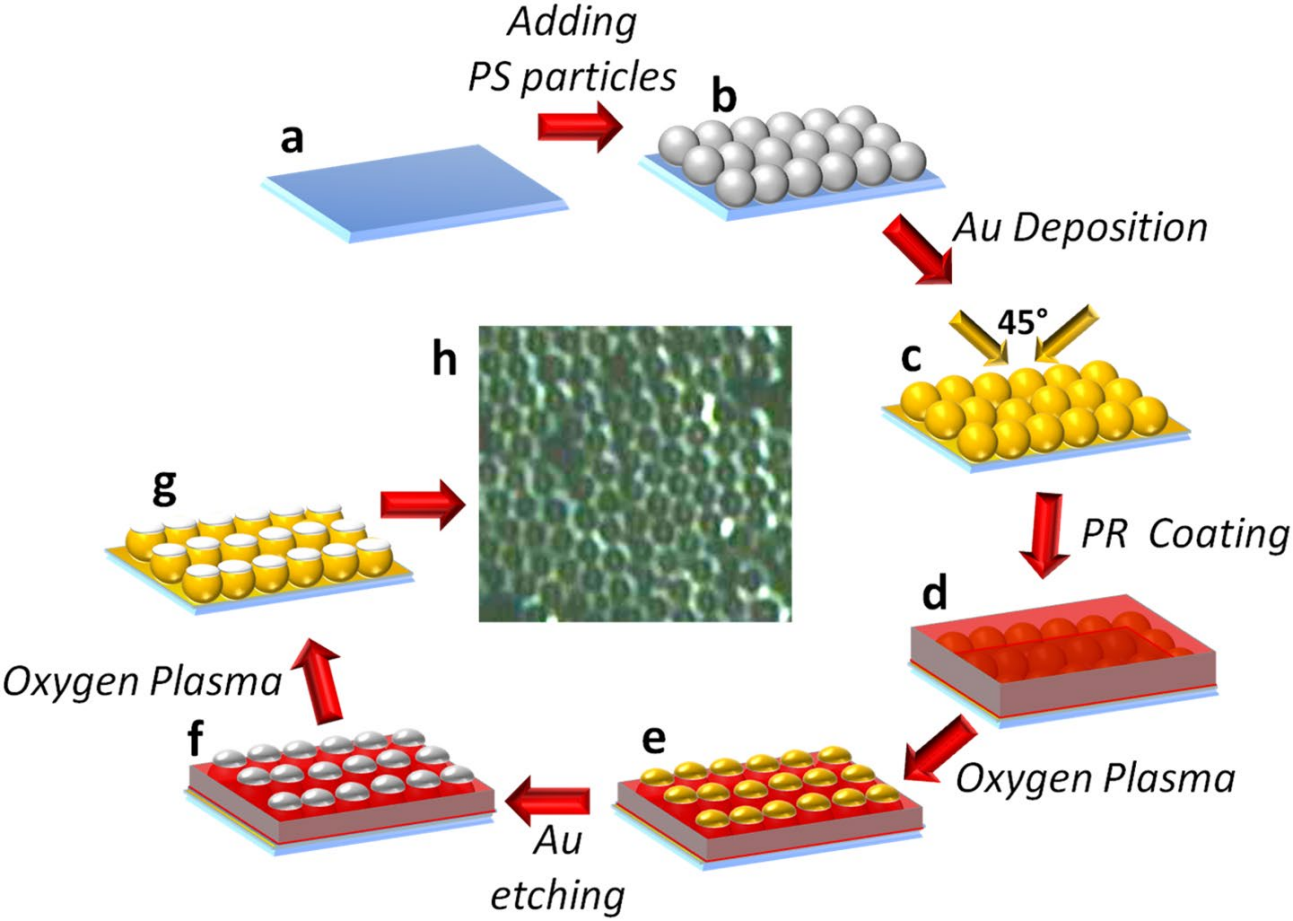
Fabrication process of the proposed gold cauldrons: (**a**) Cleaned and oxygenated glass substrate, (**b**) dispersing polystyrene spheres, (**c**) conformal coating of gold layer, (**d**) spin coating of the photoresist layer, (**e**) partial plasma ashing of photoresist to expose the top part of the particles, (**f**) etching the top part of the gold shell, and (**g**) complete removal of the residual photoresist and the inner polystyrene particle by oxygen plasma, to achieve completed gold cauldrons. (**h**) Optical top image of the final gold cauldrons cluster.

The fabrication process of the proposed gold cauldrons is shown schematically in Fig. 1a–g. It is notable that the dispersion method was used to synthesize PS spherical particles with average radius of 600 nm, which serve as the mold in the proposed fabrication process^33^. As the first step, a glass substrate is cleaned by standard RCA#1, which in combination with an oxygen plasma step can lead to an increased surface hydrophilicity of the substrate (Fig. 1a). The hydrophilic surface is essential in the next step, when the water-based suspension of PS particles is dropped on the substrate to achieve a uniform monolayer of particles. This oxygen treatment is performed by exposing the substrate to an RF oxygen-plasma with power of 200 W, at the base pressure of 395 mTorr, for about 60 s. To achieve a hexagonal closed pack (HCP) monolayer of particles, the synthesized PS suspension is drop casted on the glass substrate, and dried by a warm air flow, with normal direction to the substrate. Due to the capillary forces during drying, a uniform monolayer of PS particles with HCP structure is achieved, as shown in Fig. 1b. Then, a conformal gold layer with a thickness of about 45 nm is deposited on the PS particles, during two deposition steps by thermal evaporation, each with an incident angle of 45° with respect to the normal vector (Fig. 1c). Now, a diluted photoresist solution is spin coated on the sample to cover the PS particles, as illustrated in (Fig. 1d). To dilute the photoresist solution, we have mixed it with 2-propanol by ratio of 1:1, and placed the solution on a magnetic stirrer for 10 min. After baking the coated sample at 50 °C for 10 min, the sample is exposed to an RF oxygen plasma for partial ashing of the resist layer. This oxygenation with a precise control on the plasma time duration can reduce the resist thickness, leading to partial exposure of the particles via the resist layer (Fig. 1e).

It should be noted that the final aperture size of the gold cauldrons, which can affect the plasmonic behavior of the structures, is controllable by time duration of this plasma step. The longer plasma time duration, the larger aperture of the achieved gold cauldrons. Other conditions of this ashing step, such as plasma power and base pressure, are similar to the previous oxygenation treatment. Then, the samples are baked at 125 °C for 25 min to cure the photoresist, after which the exposed top part of the gold layer on the particles is removed by wet etching (Fig. 1f). To achieve the final 3D gold cauldrons, the remained photoresist layer and the inner PS particles should be removed by a similar oxygen plasma step, with a long time duration of 14 min (Fig. 1g). Figure 1h shows the top optical image of the fabricated structures, wherein the successful opening of the gold cauldrons are observable.

**Figure 2:**
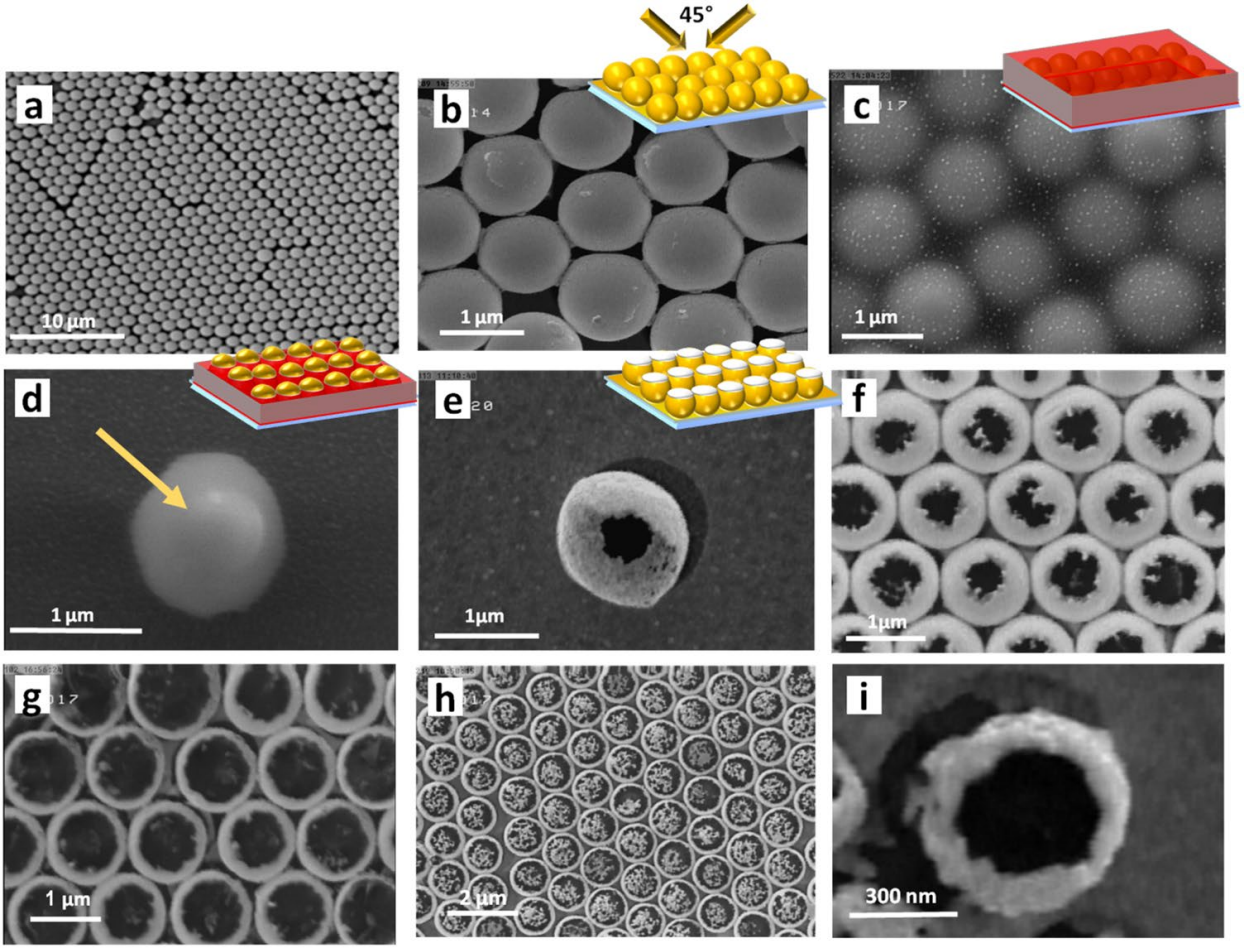
(**a**–**f**) SEM images, corresponding to different steps of the fabrication process. (**a**) HCP monolayer of PS particles. (**b**) Conformal deposition of gold layer on the PS particles. (**c**) PS particles embedded in the photoresist layer. (**d**) Partial ashing of photoresist layer and exposure of the top part of a PS particle. (**e–h**) Complete removal of the photoresist layer and the inner PS particle, and realization of a single gold cauldron (**e**), and cauldrons HCP clusters with different opening radii (**f**–**h**). (**i**) Realization of a single gold cauldron with smaller dimensions.

Figure 2a–f exhibit the SEM images corresponding to some fabrication steps, described in Fig. 1. The insets represent the corresponding structures, schematically. Figure 2a displays the SEM image of the achieved HCP monolayer of the PS particles on the glass substrate, while Fig. 2b shows the spherical PS particles with radius of 600 nm, after successful conformal deposition of gold layer. Figure 2c shows the PS particles, embedded completely in a thick layer of photoresist. Part (d) illustrates an individual PS particle after partial ashing of the photoresist, wherein the exposed top part of the particle is observable and is highlighted by an arrow. The remained photoresist layer can protect the unexposed part of the gold-coated PS particle in the next gold etching step. Part (e) in this figure shows a single gold cauldron after complete removal of the inner PS particle and the photoresist layer. Moreover, parts (f) and (g) in this figure present closed packed gold cauldrons clusters with smaller and larger openings, respectively. The observed different opening sizes are achieved due to different time durations of photoresist ashing step, as described in Fig. 1e. Part (h) in Fig. 2 proves the realized HCP monolayer of gold cauldrons over an area of about 14 × 10 µm^2^. Moreover, to prove that the presented technique enables achieving gold cauldrons with smaller radius, we have reduced the size of our synthesized PS particles by adding an extra oxygen ashing step with long time duration, before gold deposition (Fig. 1c), and repeated the rest of the fabrication process similarly. This extra ashing step reduces the radius of the PS particles, while increasing the interspacing between the particles. Figure 2i illustrates SEM image of an achieved smaller gold cauldron with radius of about 300 nm. It is observable that the achieved smaller gold cauldron lacks a perfect and symmetric spherical shape and circular opening, and also suffers from considerable surface roughness. These drawbacks are originated from the extra over exposure of PS spheres to oxygen plasma, and can be easily overcome by avoiding this step and begin the process with small PS spheres. It should be noted that we have used larger cauldrons with nearly spherical shape and negligible roughness (similar to Figure (e, f)) for our plasmonic tweezing investigations, in the rest of paper.

Here, we proved successful realization of gold cauldrons clusters with an overall processing control on the average dimensions of the cauldrons. The presented approach benefits from low cost and capability of realizing cauldrons clusters in large area and with high throughput.

## Plasmonic characterization

### Simulations

**Figure 3:**
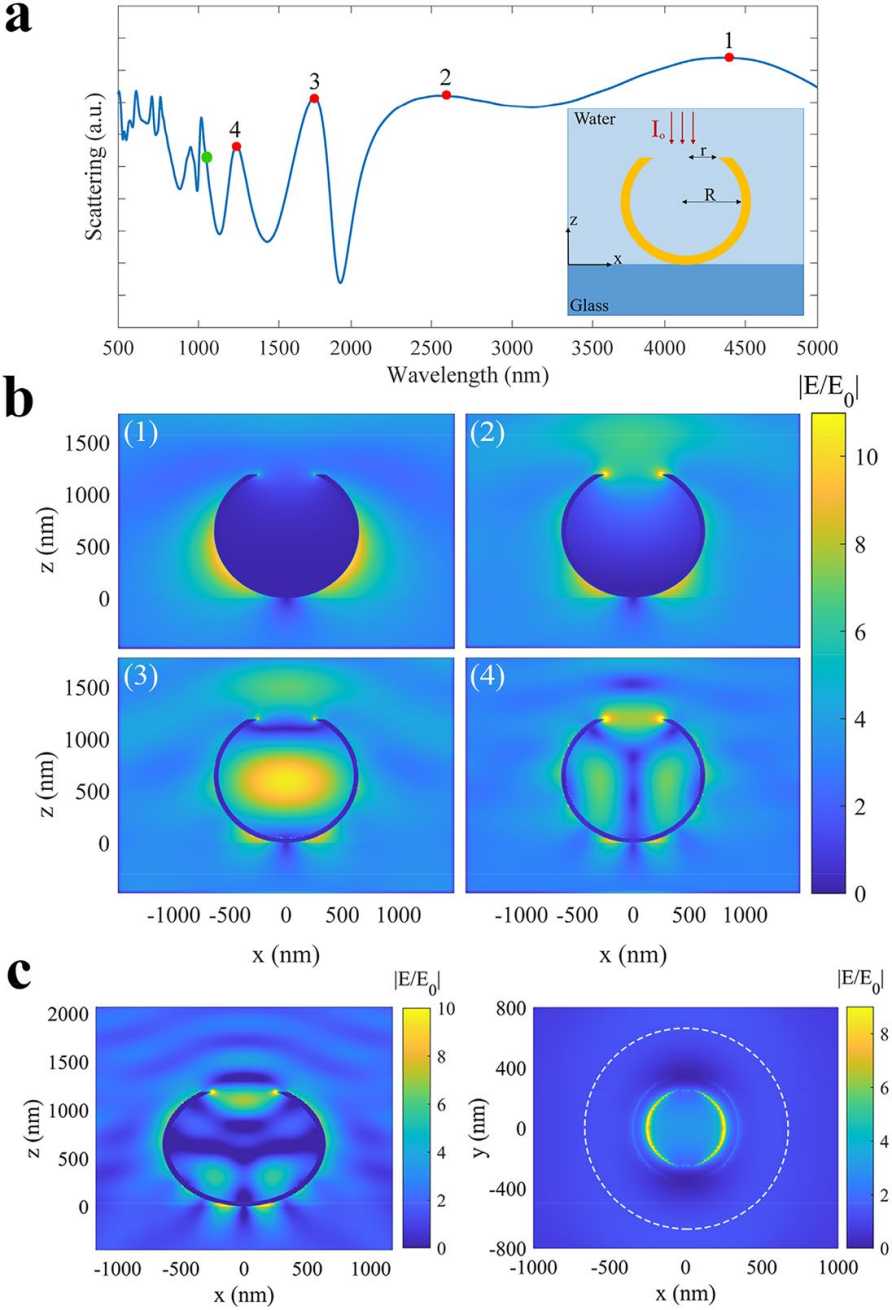
(**a**) Scattering spectrum calculated for a single gold cauldron with *R* = 600 nm and *r* = 250 nm, as shown schematically in the inset. The red points show the plasmonic resonances in the scattering spectrum, while the green point shows *λ* = 1064 nm. Absolute field distributions in the: (**b**) *x–z* plane for: (1) *λ*_*1*_ = 4400 nm, (2) *λ*_*2*_ = 2650 nm, (3) *λ*_*3*_ = 1850 nm, (4) *λ*_*4*_ = 1240 nm. (**c**) Cross (*x–z* plane) and top (*x–y* plane) views of the normalized field distributions for a single cauldron at *λ* = 1064 nm.

To elaborate the plasmonic behavior of the presented gold cauldrons, first we simulated a single gold cauldron with radius of *R* = 600 nm, the aperture radius of *r* = 250 nm, and the gold thickness of 45 nm, when illuminated by an *x*-polarized plane wave perpendicularly, as shown schematically in the inset of Fig. 3a. Here, we use three dimensional finite difference time domain (FDTD) method to calculate the backscattered spectrum of a gold cauldron on a glass substrate, while surrounded by water. The calculated scattering spectrum is shown in Fig. 3a, in which red points show the scattering peaks at: *λ*_*1*_ = 4400 nm, *λ*_*2*_ = 2650 nm, *λ*_*3*_ = 1850 nm, and *λ*_*4*_ = 1240 nm, corresponding to the first four plasmonic modes of a gold cauldron. Part (b) presents the absolute field distributions, normalized to the input source field (|E/E_0_|), for each mode in the *x–z* plane, which are numbered in accordance with the corresponding plasmonic peaks. It can be observed that the first four modes of surface plasmon polaritons (SPPs) are excited on the sidewalls of the gold cauldrons, at *λ*_*1*_, *λ*_*2*_, *λ*_*3*_, *λ*_*4*_. In fact, edge reflection of the SPPs from the aperture of the cauldron has led to emergence of a standing plasmonic wave for each plasmonic mode in Fig. 3b ^34–36^. Thus, an integer multiply of *λ*_*SPP*_/2 appears on the side walls of the gold cauldron for the first four plasmonic modes, wherein *λ*_*SPP*_ is the plasmons wavelength. Moreover, Moreno and colleagues have proved that sharp metallic edges can focus the SPPs, and lead to a high concentration of plasmonic field, and conversion of SPPs into wedge plasmon polariton (WPPs)^37^. We believe that a similar focusing behavior at the aperture edges can be responsible for the observed concentrated plasmonic field at the aperture of the gold cauldron. These localized plasmonic fields can allow efficient plasmonic trapping of the target particles by gold cauldrons.

Since, we have used a laser source with *λ* = 1064 nm (green point in Fig. 3a) for the plasmonic tweezing experiments, here we present the cross (*x–z* plane) and top views (*x–y* plane) of the normalized field distributions for a single cauldron at *λ* = 1064 nm, as shown in Fig. 3c. The top view field distribution is presented at a vertical distance of about 10 nm above the cauldron aperture, confirming a high field concentration on the cauldron aperture. The white dashed circle shows the circumference of the cauldron in the top view.

**Figure 4:**
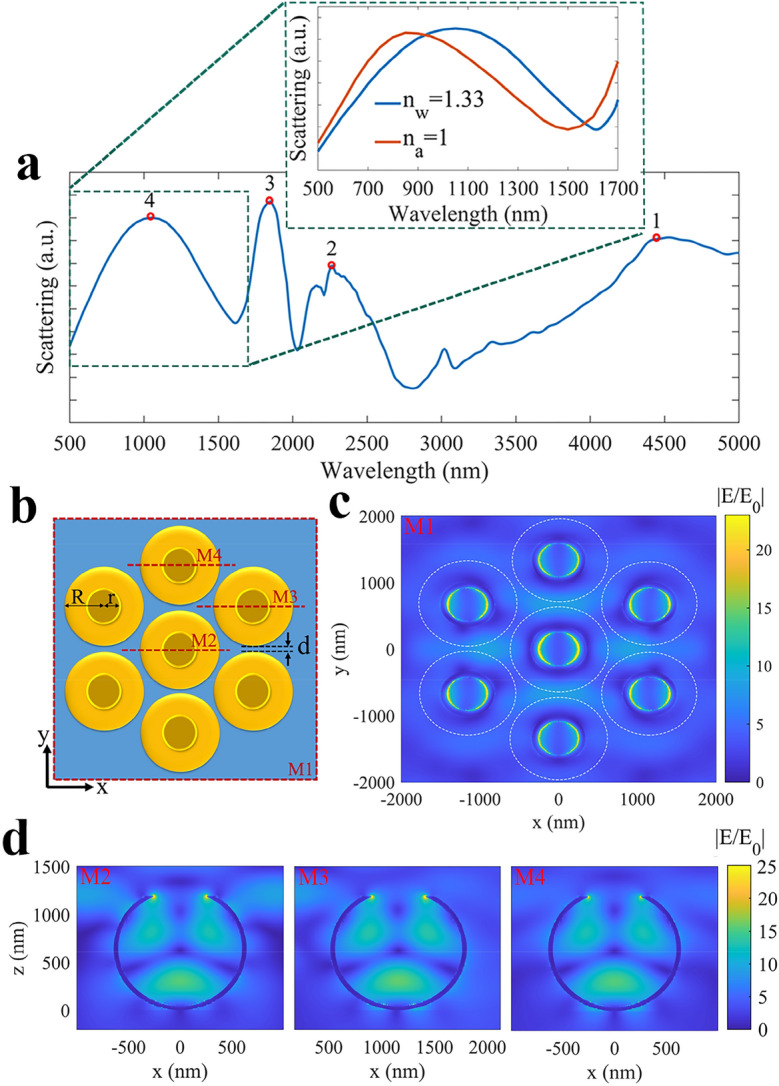
(**a**) The scattering spectrum of a cauldrons cluster with closed pack arrangement, and dimensions of *R* = 600 nm, *r* = 250 nm, and *d* = 50 nm. The inset shows the magnified scattering spectra (500–1700 nm) for the air (red spectrum) and water (blue spectrum) environments. (**b**) Top view scheme of the cauldrons cluster. (**c**) Top view of the normalized absolute field distribution in the *x–y* plane for *λ* = 1064 nm, corresponding to the red dashed rectangle (M1 in part (**b**)). (**d**) Cross views of the field distribution at cross sections M2, M3, and M4 in part (**b**), for *λ* = 1064 nm.

Then, we simulate a cluster of seven closed packed gold cauldrons with the same dimensions as the previously investigated single cauldron. The scattering spectrum of cauldrons cluster is presented in Fig. 4a, in which the achieved plasmonic peaks (shown by red circular markers) are nearly in correspondence with the scattering peaks of a single cauldron (Fig. 3a). Since, we have used a laser source with *λ* = 1064 nm for the plasmonic tweezing experiments by cauldrons cluster, we focus on the wavelength range of 500–1700 nm, and present a magnified view of the scattering spectrum in the inset. Red and blue spectra in the inset reveal the scattering spectra of the cauldrons cluster, in the air environment (*n*_*a*_ = 1) and the water environment (*n*_*w*_ = 1.33), respectively. These simulations correspond to our experimental plasmonic spectroscopy in the air medium, and our plasmonic trapping experiment in water medium. It can be observed that the air-relating plasmonic spectrum shows a wide plasmonic peak at about 850 nm, and water medium shifts the plasmonic peak to about 1050 nm. The observed water-related plasmonic spectrum proves that the gold cauldrons cluster with the presented dimensions is suitable for plasmonic trapping in the aqua environment with a laser source of *λ* = 1064 nm, which is conventional in optical tweezers. Figure 4b exhibits the top view of the investigated cauldrons cluster schematically, while their interspacing distance is *d* = 50 nm. These structural dimensions and arrangement are in agreement with the average dimensions of the gold cauldrons, which have been used for experimental plasmonic characterization and plasmonic tweezing. Figure 4c shows the plasmonic absolute normalized field distribution in the *x–y* plane at a vertical distance of about 10 nm above the cauldron apertures over the red dashed rectangle in part (b) (M1), at *λ* = 1064 nm. The dashed white circles in this figure show the circumference of the neighboring cauldrons, and it can be observed that the plasmonic field is concentrated on the cauldron apertures. Figure 4d displays the cross views of the normalized field distribution over cross sections M2, M3, and M4 in part (b), relating to the mid cauldron and two peripheral cauldrons in the cluster. Comparing the normalized absolute field distributions of a single cauldron (Fig. 3c) with a cauldrons cluster (Fig. 4c,d) at *λ* = 1064 nm, reveals that the constructive superposition of the backscattered fields from the nearby cauldrons leads to a stronger field concentration and enhanced localized field in the cluster^19^. It can be observed that the maximum value of the normalized field is increased from about 10 for a single cauldron to about 25 for the mid cauldron, and about 18 for the peripheral cauldrons in the cluster.

### Measurements

**Figure 5:**
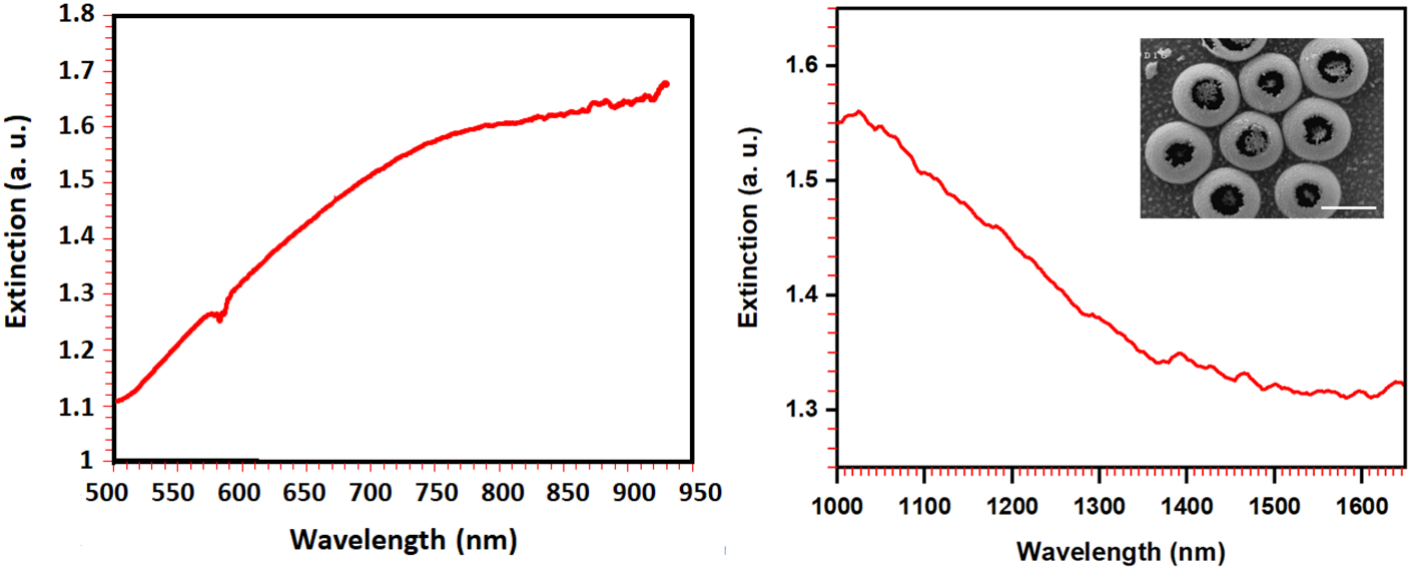
The extinction spectra of the fabricated gold cauldrons cluster with average dimensions of about *R* = 600 nm, *r* = 250 nm, and *d* = 50 nm, in the visible (left) and near infrared (right) ranges. The inset in the near infrared spectrum shows the SEM image of the investigated cauldrons cluster. The scale bar in the inset is 1 µm.

Now, we investigate the plasmonic behavior of the fabricated gold cauldrons cluster by visible and near infrared spectroscopy in air medium. For this purpose, a focused beam of broadband sources is illuminated to the sample, and the transmitted beam is fed to fiber-coupled visible and near infrared spectrometers, subsequently. The spot size of the focused source beam in this experiment can excite a cluster of a few gold cauldrons, similar to Fig. 4b. Figure 5 illustrates the extinction spectra of the fabricated gold cauldrons with average dimensions of about *R* = 600 nm, *r* = 250 nm, and *d* = 50 nm in the visible and near infrared ranges. The inset in the infrared spectrum shows an SEM image of a sample gold cauldrons cluster. It can be observed that the experimental spectroscopy results are in overall acceptable agreement with the inset spectrum in Fig. 4a. It is notable that the tolerance in the structural parameters of the fabricated cauldrons are obviously responsible for the observed differences between the simulation and experimental spectroscopy results. It should be noted that both experiment and simulation confirm that the presented gold cauldrons are suitable for plasmonic tweezers application, utilizing a Nd:YAG laser source of *λ* = 1064 nm.

## Plasmonic enhanced optical tweezers with gold cauldrons

**Figure 6:**
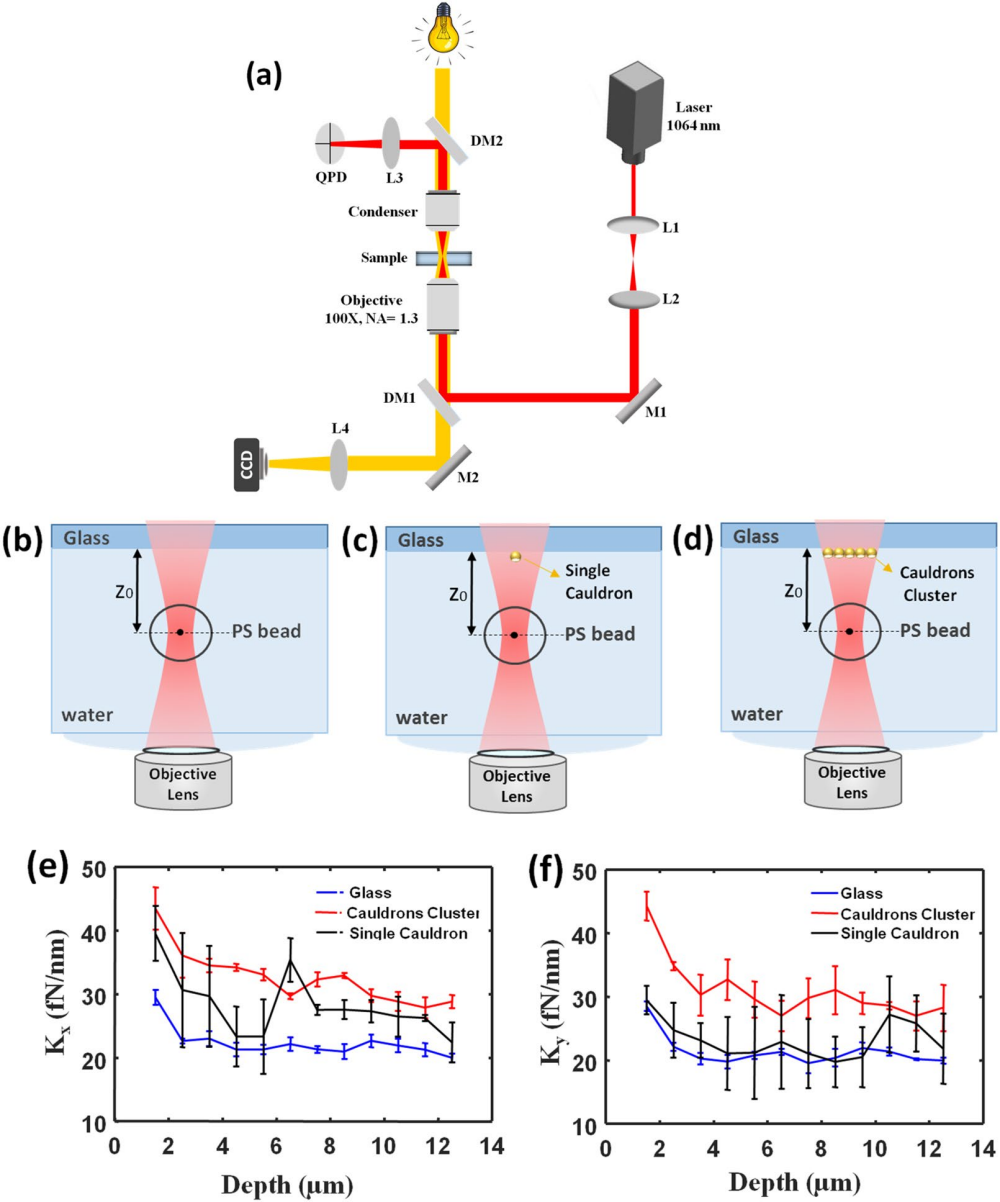
(**a**) The scheme of the optical tweezers setup. A near infrared laser (*λ* = 1064 nm) is used for trapping experiments. A QPD measures the position of the trapped bead by back-focal-plane detection, which is amplified before recording it with a computer. *L* Lens, *M* Mirror, *DM* Dichroic Mirror, *QPD* Quadrant Photodiode. A bright illumination is used for the microscopy and a CCD is used for imaging the trapped particle and the structure. The scheme of PS trapping below the: (**b**) bare glass, (**c**) single gold cauldron, (**d**) and cauldrons cluster. Variation of the measured average trap stiffness as a function of depth below the bare glass, single cauldron, and cauldrons cluster along: (**e**) *x*-direction, (**f**) and y-direction.

In order to investigate the functionality of the realized gold cauldrons to enhance the optical tweezers force, first we study the trap stiffness modulation of an optically trapped particle at the vicinity of our plasmonic structures. Using a focused laser beam to a diffraction limit, we trapped a PS particle with radius of 500 nm at the bottom of the microfluidic chamber. Figure 6a shows the scheme of the optical tweezers setup^19,38–41^. It consists of a Nd:YAG laser source (CW, *λ* = 1064 nm, Coherent) a telescope system, and an inverted microscope (Olympus, IX 71). The beam expander (L1 and L2) expands the laser beam, and it is directed to the microscope after reflection from M1 and DM1. The laser beam is tightly focused into the sample using a water immersion objective lens (Olympus UPlanSApo, 60x, NA = 1.2). The laser power is about 33 mw. The sample chamber made by the plasmonic substrate, as top surface, a coverslip, as the bottom surface, and double-sided sticky tape with a thickness of ∼ 100 μm as a spacer, is mounted on a piezo stage (Physik Instrumente, PI-527.2 cl). Once the particle was trapped in the vicinity of the plasmonic structures, the positional signal from the quadrant photodiode (QPD) was recorded as time series, using a custom-made LabView program. The distance between the trapped particle and the substrate can be axially changed by moving the position of the focused laser beam, while the positional signal from photodiode was recorded every one micrometer. Thus, we can investigate the modulation of the optical trap stiffness, when the particle is trapped below three different zones of the sample: bare glass, single gold cauldron and gold cauldrons cluster, as shown schematically in Fig. 6b–d, respectively. For this purpose, we measure the lateral trapping stiffness (*k*_*x*_, *k*_*y*_), when the particle is trapped at different depths (*z*_*0*_ values as shown in Fig. 6b–d below each zone. Each measurement recorded for ∼3 s, and is repeated three times for three similar particles at each region. The measured average trap stiffness values are presented in Fig. 6e,f, while the error bars display the tolerance in the measurements. It can be obviously observed that for both single and cluster, the trap stiffness is higher than the bare glass at a similar depth, which is attributed to the excitement of surface plasmons of the gold cauldrons. In other words, the plasmon excitement leads to a local field enhancement at the vicinity of the gold structures, resulting in an enhanced gradient force and trap stiffness, consequently. Moreover, the measured trap stiffness of the cluster is more than the measured value for the single structure, due to the superposition of the plasmonic fields of the adjacent gold cauldrons. This observation is in accordance with the simulated field distributions in Fig. 4c,d and Fig. 3c that proved higher localized fields at the aperture of a cauldron in a cluster, in comparison with a single cauldron. One other remarkable agreement between theory and experiment is the effect of laser polarization on increasing the optical stiffness. As can be seen, trapping stiffness shows higher enhancement along the direction of the laser polarization (*x-*direction) at the vicinity of a single structure, comparing with the trapping stiffness along *y-*direction. The other worthy point in Fig. 6e,f is that both trap stiffness values are reduced significantly by increasing the trapping depth, which is attributed to the inherent short range behavior of the plasmonic fields. However, considering that the excited plasmonic fields are expected to decay exponentially away from the cauldrons, deviation of the presented Plasmon-induced trapping stiffness values from those of the bare glass at depth values higher than 3 µm, is attributed to some other phenomena, such as thermal or optical origins. When the laser beam is focused in front of a gold layer, gold acts as a metallic mirror, and changes the intensity distribution near the laser focus point. It behaves as an additional external force that changes the equilibrium position of the realized optical trap^42^, or produces a bistability for the trapped particles position^43^. Another reason is probably the effect of heat generated by plasmonic structures at resonance, which can lead to a temperature gradient field in water, and can modify the measured trapping stiffness, even at longer distances from the cauldrons^44^. Moreover, in plots of Fig. 6e,f there are some data points that don’t follow the overall trend. This is probably due to the jump of the particle in the potential wells close to each other, which can be caused by aberration or light reflection from the gold structures.

To evaluate the trapping stiffness in the latter measurement, a stochastic method was used^45^. For this purpose, the power spectrum is derived by $p\left(f\right)=\frac{<{\left|x\left(f\right)\right|}^{2}>}{L}$, wherein *x(f)* is the Fourier transform of the recorded position of the trapped particle as a function of time (*x(t)*), and *L* is the number of particle position sampling records. The calculated power spectrum of the trapped particle is fitted by a Lorentzian function of $p\left(f\right)=\frac{{k}_{B}T}{2{\pi }^{2}\gamma ({f}^{2}+{f}_{c}^{2})}$, from which the trap stiffness (*k*) is then achieved from $k=2\pi \gamma {f}_{c}$ ^45–47^. Here, *γ* is the Stokes drag coefficient, which is derived from $\gamma =6\uppi \eta r$, *fc* is the corner frequency, *k*_*B*_ is the Boltzmann constant, *T* is the temperature, *η* is the fluid viscosity, and *r* is the radius of the trapped particle.

Then, we present the average Plasmon-enhanced trap stiffness at the vicinity of the cauldrons cluster in comparison with the bare glass in Table 1, to clarify the relative trapping enhancement value (*∆k/k*(%) = (*k*_Cauldrons-Cluster_-*k*_Glass_)/*k*_Glass_). This table illustrates that plasmon-induced trap stiffness is enhanced about 60.6% and 55% along *x* and *y* directions at *z*_*0*_ = 1.5 µm, while these enhancements are decreased for *z*_*0*_ = 12.5 µm, revealing the near field enhancement of the plasmon field.

**Table 1:** The measured average *k* for *z*_*0*_ = 1.5 µm and *z*_*0*_ = 12.5 µm, above the bare glass and gold cauldrons cluster.

Tweezers configurations				
Direction	z_0_ (µm)	Bare glass *k* (fN.nm^−1^)	Cauldrons cluster *k* (fN.nm^−1^)	*∆k/k* (%)
*X*	1.5	29	46.6	60.6
	12.5	20	28.9	44.5
*Y*	1.5	28.5	44.2	55
	12.5	20	28.3	41.5

## Plasmonic trapping by the gold cauldrons cluster

### Simulations

**Figure 7:**
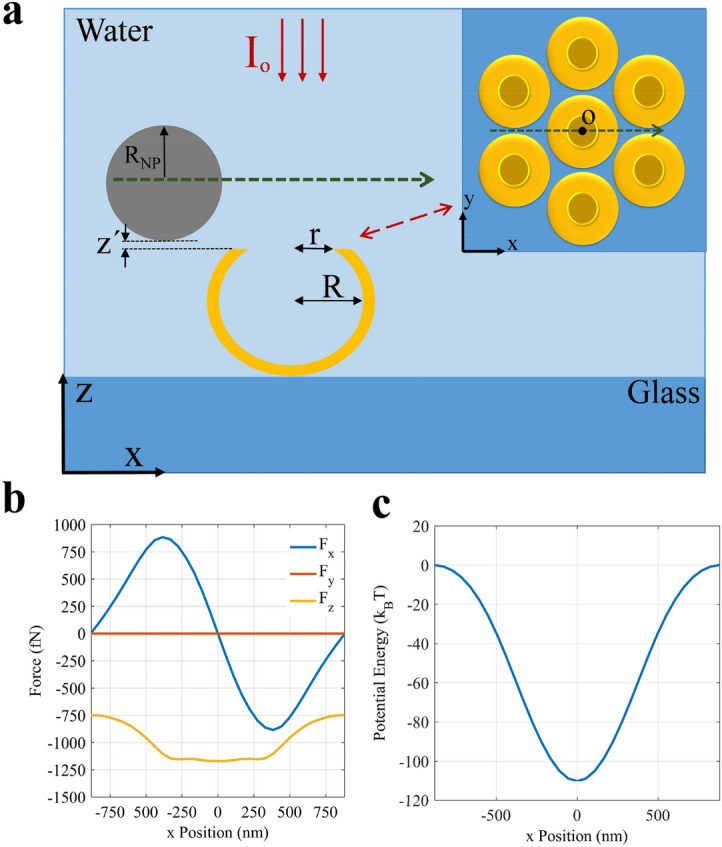
(**a**) A PS particle of radius *R*_*NP*_ = 500 nm that is passing above a cauldrons cluster, along the green dashed arrow, at *z'* = 10 nm in the x–z plane. The inset shows the corresponding view in the x–y plane. (**b**) The exerted plasmonic force components, (**c**) and the potential energy of the PS particle.

To study the trapping functionality of the presented gold cauldrons, we calculate the plasmonic forces exerted on the PS particles with radius of *R*_*NP*_ = 500 nm, in the water medium. A laser source of *λ*≈1064 nm, with intensity of *I*_*s*_ = 33 mW/μm^2^ has been used for plasmon excitement, in our simulations. Moreover, we have assumed the vertical position of the PS particle at 10 nm above the center of a cauldron aperture (*z’* = 10 nm), and moved the particle along the green dashed arrow, as shown schematically by the cross and top views in Fig. 7a. In order to calculate the plasmonic forces exerted to the target particles, we have applied Eq. (1) to evaluate the surface integral of the time-averaged of the Maxwell’s stress tensor (**T**), numerically^48^:

1 $$<\mathbf{F}>=\frac{1}{2}\mathrm{Re}\underset{\Omega }{\overset{}{\oint }}\mathbf{T}\left(\mathrm{r},\mathrm{t}\right).\widehat{\mathbf{n}}\mathrm{ds}$$

2 $$\mathbf{T}\left(\mathrm{r},\mathrm{t}\right)=\upvarepsilon \mathbf{E}\left(\mathrm{r}\right)\otimes {\mathbf{E}}^{\mathbf{*}}\left(\mathrm{r}\right)+\upmu \mathbf{H}\left(\mathrm{r}\right)\otimes {\mathbf{H}}^{\mathbf{*}}\left(\mathrm{r}\right)-\frac{1}{2}(\upvarepsilon {\left|\mathbf{E}\left(\mathrm{r}\right)\right|}^{2}+\upmu {\left|\mathbf{H}\left(\mathrm{r}\right)\right|}^{2})$$

wherein **T**(**r, t**) is the Maxwell stress tensor and is derived from Eq. (2), *ε* and *μ* are the medium permittivity and permeability, **E** and **H** are the electric and magnetic field intensity vectors, **r** and *t* represent the position vector and time, and **n** is the unitary vector normal to the surface that encloses the volume *Ω*. Moreover, the trapping potential profiles are obtained by the integral $\int {F_{x} dx}$, where *F*_*x*_ is the x-component of the plasmonic force. Part (b) and (c) in Fig. 7 show the calculated plasmonic forces and potential energy, respectively.

It is observable in part (b) of this figure that the z-component of the plasmonic force (*F*_*z*_) is negative above the cauldron, revealing that the PS particle is attracted towards the cauldron. It is notable that y-component of the plasmonic force is zero (*F*_*y*_ = 0), due to the structural symmetry of the cluster with respect to the x-polarization of the incident light. However, the sign of *F*_*x*_ switches at the center of the cauldron, so that leads to potential well along x-direction for the PS particle. Figure 7c displays that the depth of the potential well exceeds − 100*k*_*B*_*T*, revealing a completely stable plasmonic trapping. Since the potential depth has a nearly linear relation with the laser source intensity^24^, we can deduce that a tenth of the applied laser intensity is sufficient for stable trapping of the PS particle with *R*_*NP*_ = 500 nm. Here, we assume that the minimum required potential depth for achieving stable particle trapping is about 10*k*_*B*_*T*^49^.

### Experiment

**Figure 8:**
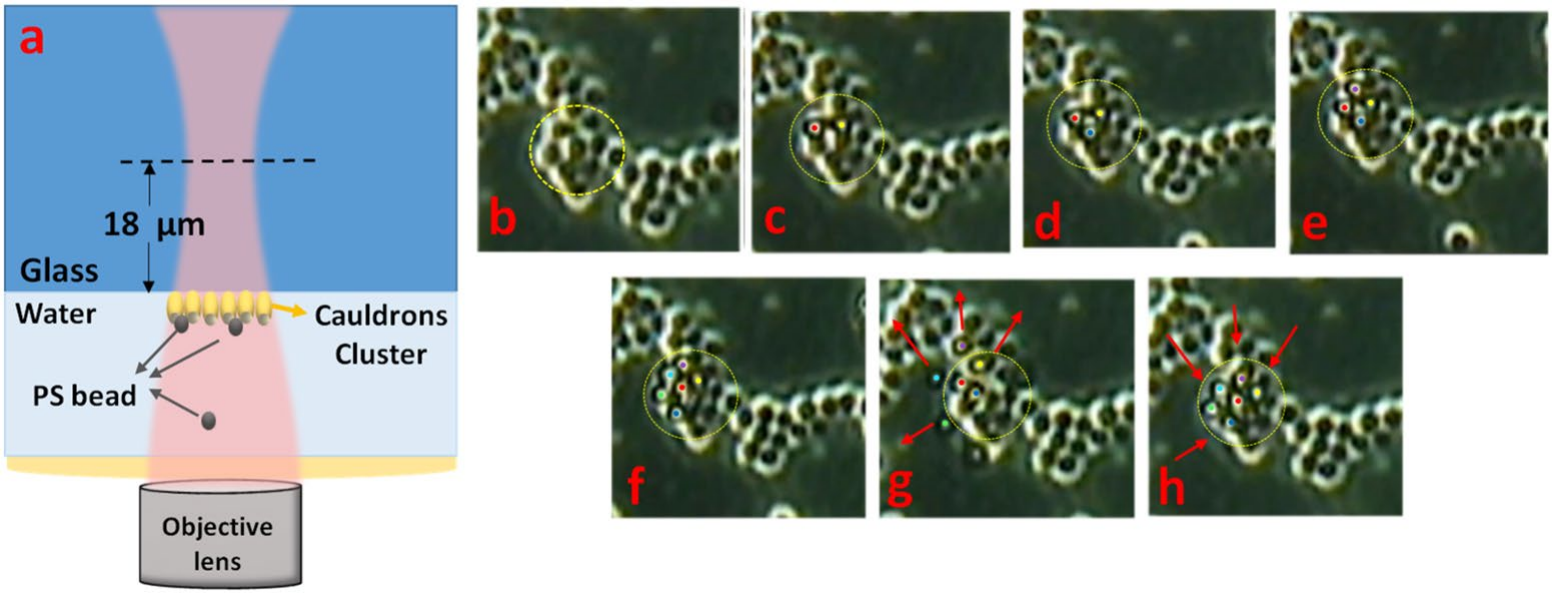
(**a**) The optical configuration used for the plasmonic trapping experiment. (**b**–**h**) Different time frames of plasmonic trapping of PS particles with *R*_*NP*_ = 500 nm. The laser source is ON during parts (**b**–**f**), turns OFF in part (**g**), and again turns ON in part (**h**). The fixed location of the laser spot is shown by dashed circles, and the target PS particles are shown by colored circular markers.

To elaborate the functionality of the fabricated gold cauldrons as a plasmonic tweezers directly, we have modified the optical configuration in Fig. 6a to weaken the gradient force of the focused laser beam well below the threshold of direct optical trapping. For this purpose, we have replaced the objective lens in the optical configuration by a variable numerical aperture objective lens (Zeiss, Plan Apo, 63x, *NA* = 0.7–1.4), which is set to the lowest possible numerical aperture with the same magnification, *NA* = 0.7. We also have focused the laser beam at about 18 μm behind the plasmonic structures, to provide a larger area irradiated by the laser, as shown in Fig. 8a schematically. The laser source power is the same as Sect. 4, however, it should be noted that the radius of the illuminated area is ≈10 µm, and the intensity could be roughly estimated ≈0.1 mW/µm^2^. The modified configuration does not lead to any direct optical trapping of the PS target particles with the radius of *R*_*NP*_ = 500 nm, in the absence of the gold structures (on the bare glass). However, by moving the beam toward the gold cauldrons, successful plasmonic trapping of the particles is observable, as illustrated at different time frames of parts (b–h) in Fig. 8. The gold cauldrons are observable and the position of the laser spot is shown by a dashed circle in this figure, wherein the number of trapped PS particles is increased during time from part (c) to (f). Random movement of the target particles and stable trapping condition on the excited gold cauldrons at the laser spot position leads to increment of the trapped particles during the time that the laser source is on (parts b–f). The trapped particles in the vicinity of the cauldrons cluster are highlighted by colored circular markers. It can be observed in part (f) that six trapped PS particles follow the closed pack pattern of the plasmonic cauldrons, which are responsible for the observed successful plasmonic trapping. Then, part (g) shows that the trapped particles are released by turning the laser off, and are moving away. However, by turning the laser on, the particles are trapped again and rearranged below the gold cauldrons in a closed pack pattern, as indicated in part (h). The recorded movie of the plasmonic trapping, relating to this figure is presented as Visualization 1 in the supplementary data of this manuscript. It should be noted that the optical tweezers setup is implemented on a transmitted-light microscope, and the gold layer on the plasmonic substrate prevents sufficient illumination of the particles to provide an ideal image. However the movement of free and trapped particles could be well recognized in Visualization 1. Comparing the applied laser power in this plasmonic trapping experiment, with the reported laser power required for plasmonic tweezing a 1 µm PS bead in the vicinity of a double antenna^50^, or nano-aperture arrays^51,52^ entitles the presented gold cauldrons as reasonable candidates for plasmonic tweezing.

## Summary

We introduced a controllable fabrication of gold cauldrons, as 3D cavity-like plasmonic structures, using microsphere lithography method. We investigated the plasmonic behavior of the fabricated cauldrons clusters by simulation and near infrared spectroscopy, and proved high plasmonic field localization on the apertures, owing to the additive fields of the neighboring cauldrons. To investigate the behavior of the realized structures in plasmonic tweezers application, first we studied the effect of presence of the plasmonic structures on the trapping stiffness via a conventional optical tweezers. We proved that the plasmon-induced trap stiffness is highest at the vicinity of the cauldrons cluster, comparing with the single cauldron and the bare glass substrate, which is attributed to the additive effect of the plasmonic fields of the neighboring cauldrons. To elaborate the direct plasmonic trapping functionality of the presented cauldrons cluster we calculated the plasmonic force, exerted to the PS particles, revealing an efficient plasmonic trapping capability. Moreover, we proved successful direct plasmonic tweezing by the gold cauldrons cluster, via a modified optical tweezers configuration. In summary, the presented gold cauldrons benefits from a simple and low cost fabrication process, allowing controllable structural dimensions and plasmonic characteristics, consequently. The achieved plasmonic gold cauldrons show a high localized field, suitable for plasmonic tweezing, making them attractive candidates for emerging optophoresis LOC systems.

## Supplementary information

Supplementary Video 1.
